# Healthy futures online: A rights-based perspective on digital health literacy for children and youth

**DOI:** 10.1371/journal.pdig.0000946

**Published:** 2025-08-04

**Authors:** Jia Lin, Maria Fernanda Vargas Herrera, Alice Sofia Rubio, Taty Diego, Marie-Eve Turcotte, Alexanne Dumas, Malika Saher, Bertrand Lebouché, Esli Osmanlliu

**Affiliations:** 1 Department of Family Medicine, Faculty of Medicine and Health Sciences, McGill University, Montréal, Québec, Canada; 2 Centre for Outcomes Research and Evaluation (CORE), Research Institute of the McGill University Health Centre, Montréal, Québec, Canada; 3 Garage à Musique, Fondation Dr Julien, Montréal, Québec, Canada; 4 Institut de Pédiatrie Sociale en Communauté, Fondation Dr Julien, Montréal, Québec, Canada; 5 Droit Intégré Pour les Enfants, Fondation Dr Julien, Montréal, Québec, Canada; 6 Chronic Viral Illness Service, Division of Infectious Diseases, Department of Medicine, McGill University Health Centre, Montréal, Québec, Canada; 7 Department of Pediatrics, Division of Pediatric Emergency Medicine Montreal Children’s Hospital, McGill University Health Centre, Montréal, Québec, Canada; CHOC: Children's Hospital of Orange County, UNITED STATES OF AMERICA

*Jane, 15-years old, loves scrolling through social media for funny reels, stories of her friends, and helpful tips. One day, she sees a video about a “magic diet” that doesn’t look that healthy at all. Another video shows “quick fixes” for feeling sad or anxious. Jane knows the internet is full of exciting things, but reminds herself – not everything online is true.*

We increasingly live in a digitalized world and today’s children and youth are growing up immersed in digital technologies. With access to smartphones, up to 95% of North American youth aged 13–17 use at least one social media platform, relying on them for community, information, and creative expression [1,2]. However, the plethora of health-related information online can be concerning, especially with the rise of artificial intelligence (AI). For example, TikTok’s algorithm-driven content amplification can expose youth to highly sensitive or biased health content [3], often presented without oversight. In an era of ubiquitous digital content, how can children and youth discern trustworthy health information?

## Transdisciplinary collaborative

We are a transdisciplinary collaborative working across fields of pediatric health, social pediatrics, youth engagement, and children’s rights, including physicians, researchers, and youth collaborators. Our vision of transdisciplinarity reflects community social pediatrics (CSP), uniting academic, professional, and experiential expertise to enable children and youth equal credibility in co-constructing knowledge and action [4]. As part of this work, we issued a call for dialogue with youth at a CSP center about digital health, inviting them to co-author and contribute their unique perspectives. Together, we present the relevance of a rights-based perspective to digital health literacy that recognizes children and youths’ rights to safe digital spaces and necessary skills to assess health information as informed participants.

## Children and youths’ digital health landscape

Adolescence is a pivotal development stage marked by physical, emotional, and social changes. Social media serves as an appealing and anonymous resource, allowing children and youth to gain health-related information and knowledge. Research suggests that youth value the accessibility and relatability of health information from social media, yet may lack the critical literacy skills to navigate this landscape [5]. The proliferation of AI and mis/disinformation on platforms like TikTok, which often prioritize engagement over accuracy, present a challenge for children and youth trying to make sense of their health, alongside issues of privacy and data sharing [6]. False health information could lead to adopting unhealthy habits, developing skewed self-perceptions, or trivializing mental health struggles, among other impacts [7]. Further, digital experiences and risks are not uniform. Access, moderation, and digital literacy often vary across socioeconomic backgrounds, ethnicity, and regions [2], introducing disparities in children and youth’s digital health exposure.

## A rights-based perspective

The digital age necessitates a shift in digital health literacy for children and youth. We argue that children and youth have the right to safe online spaces and be equipped with the skills necessary to navigate digital health content responsibly. This call is not for increased access, but for safe and equitable participation. From the perspectives of the youth co-authors, their voices have often been overlooked in discussions about digital health literacy, despite their direct experiences. For them, access to accurate health information and the development of skills to critically assess such information represents an integral aspect of their autonomy and agency. This argument aligns with the United Nations Convention on the Rights of the Child (UNCRC), which states children’s rights to information (article 17), participation (article 12), and health (article 24), among other rights [8], as well as UNICEF’s approach to digital health [9].

This rights-based perspective encompasses three core themes:

1. **Right to safe online spaces**: children and youth should have access to trustworthy online health information.
2. **Right to digital health literacy skills**: they should be equipped with skills to discern online health information, ensuring they are well-informed participants in knowledge and decision-making.
3. **Right to digital sobriety**: they should have the choice to limit digital exposure, acknowledging that excessive content can negatively impact mental health.

Recognizing digital health literacy as a right shifts the paradigm from an over-protectionist model to one that affirms children and youths’ capacity for active, informed participation. Policy responses often emphasize control and restriction in response to perceived risks, thereby overlooking children and youths’ potential to engage critically with digital information [10]. An exclusively protectionist model can undermine children and youths’ fundamental rights, particularly those associated with self-determination, and can result in interventions that are not in their best interests [11,12]. While adult guidance remains essential, support must balance children and youth’s growing agency and critical thinking. Promoting safer online spaces may involve regulatory safeguards, such as greater transparency in AI health content that details how information is curated, paired with digital literacy training to strengthen the epistemic autonomy of children and youth in digital contexts [6].

Rights-based approaches to children’s digital media practices are gaining attention, providing a framework for research, policy, and initiatives that balance protection while enabling children and youth to maximize the opportunities and benefits of connectivity [10]. Nevertheless, regional and national laws can shape how these rights are operationalized [13], underscoring the need for a collaborative rights-based framework with local governance.

## CSP and digital health literacy

CSP, as an integrated model of social medicine, can enable healthcare professionals to develop proximal and long-term connections with children and families, providing a favorable context for advocating for safer online spaces and digital health literacy [4]. CSP centers also host rights committees where children and youth can exercise their rights and develop critical thinking [4], especially in AI-mediated spaces. Research supports literacy training in enhancing resilience to misinformation by fostering critical thinking, especially among youth [14]. Central to this approach is the co-design of digital health literacy innovations with children and youth [15]. In an example of frugal innovation that promotes youth-led digital sobriety, one of our co-authors improved participation by inviting her peers to place phones in her multi-pocketed jeans during their music classes. Local, nuanced, sustainable, youth-initiated interventions such as this one can inform broader policy changes.
